# LGCA-VHPPI: A local-global residue context aware viral-host protein-protein interaction predictor

**DOI:** 10.1371/journal.pone.0270275

**Published:** 2022-07-05

**Authors:** Muhammad Nabeel Asim, Muhammad Ali Ibrahim, Muhammad Imran Malik, Andreas Dengel, Sheraz Ahmed

**Affiliations:** 1 Department of Computer Science, Technical University of Kaiserslautern, Kaiserslautern, Germany; 2 German Research Center for Artificial Intelligence GmbH, Kaiserslautern, Germany; 3 National Center of Artificial Intelligence, National University of Sciences and Technology, Islamabad, Pakistan; Parc de Recerca Biomedica de Barcelona, SPAIN

## Abstract

Viral-host protein protein interaction (PPI) analysis is essential to decode the molecular mechanism of viral pathogen and host immunity processes which eventually help to control viral diseases and optimize therapeutics. The state-of-the-art viral-host PPI predictor leverages unsupervised embedding learning technique (doc2vec) to generate statistical representations of viral-host protein sequences and a Random Forest classifier for interaction prediction. However, doc2vec approach generates the statistical representations of viral-host protein sequences by merely modelling the local context of residues which only partially captures residue semantics. The paper in hand proposes a novel technique for generating better statistical representations of viral and host protein sequences based on the infusion of comprehensive local and global contextual information of the residues. While local residue context aware encoding captures semantic relatedness and short range dependencies of residues. Global residue context aware encoding captures comprehensive long-range residues dependencies, positional invariance of residues, and unique residue combination distribution important for interaction prediction. Using concatenated rich statistical representations of viral and host protein sequences, a robust machine learning framework “LGCA-VHPPI” is developed which makes use of a deep forest model to effectively model complex non-linearity of viral-host PPI sequences. An in-depth performance comparison of the proposed LGCA-VHPPI framework with existing diverse sequence encoding schemes based viral-host PPI predictors reveals that LGCA-VHPPI outperforms state-of-the-art predictor by 6%, 2%, and 2% in terms of matthews correlation coefficient over 3 different benchmark viral-host PPI prediction datasets.

## 1 Introduction

Viral pathogens are causing millions of deaths every year around the world [1]. To date, more than 44 million people have died due to human immunodeficiency virus (HIV) [2] and hepatitis B has caused over 9 million deaths [3]. These infections afflict the hosts through convoluted interaction mechanism occurred between protein of virus cells and human cells [4]. Considering the influx of such infections, healthcare organizations of different countries are investing trillions on the deep analysis of viral-host protein protein interactions (PPI) to decode the ways viruses infect and perform their life activities within host by hijacking the immune system, to better control the viral diseases, develop anti-viral drugs to break the transmission of viral infections, and to develop antibodies to push the immune system to fight viral diseases. Comprehensive knowledge of viral host PPIs is the main driving force behind the development of viral vector gene therapies that prevent diseases by immunization and treatment by gene replacement [5]. Consequently, in-detail exploration of viral-host PPIs is critical to acquire a deeper understanding of viral pathogenesis and to develop effective preventive as well therapeutic strategies.

Initially, physical contacts between viral proteins and host cell proteins were identified by high-throughput wet experimental approaches such as yeast two-hybrid (Y2H), Tandem Affinity Purification (TAP), mass spectroscopy (MS) [6–8]. However, these experimental approaches are expensive, and finding interactions between viral and host proteins on a large scale through these approaches is not feasible [9]. Furthermore, due to cost and time constraints, these experimental approaches have been used to find viral host PPIs between species, while the molecular analysis of viral host PPIs between species remained understudied [9]. Considering the success of Artificial Intelligence (AI) in multifarious Natural Language Processing [10], and Bioinformatics tasks [11], researchers have developed various AI based computational approaches for viral-host PPI prediction [12–18].

**Fig 1:**
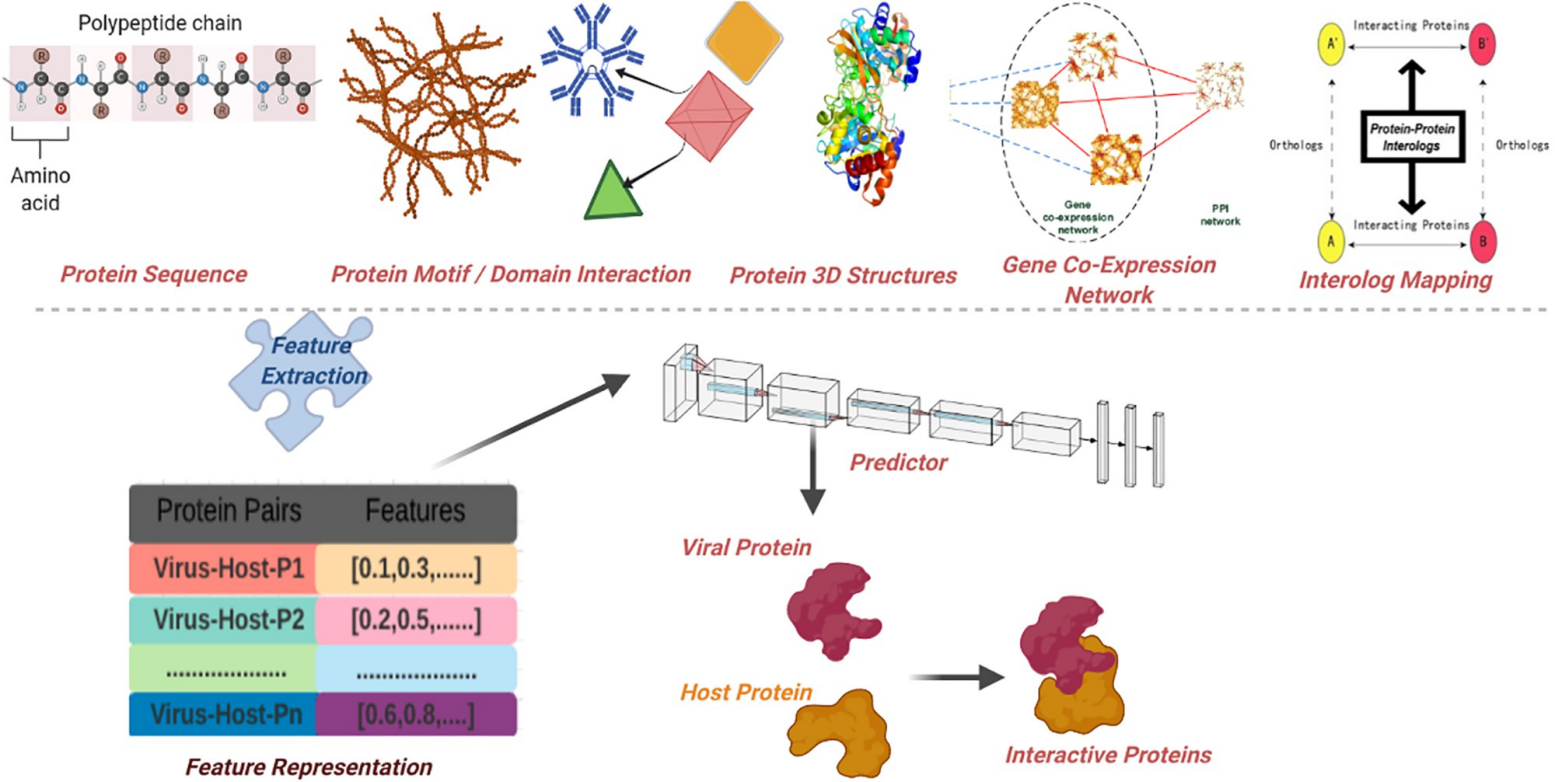
A generic categorization of different computational virus-host protein protein interaction predictors.

To accurately discriminate interactive viral-host protein sequence pairs from non-interactive ones, existing viral-host PPI prediction approaches have utilized a variety of genetic, structural, and biochemical features [19–21]. Based on the type of biological features, existing predictive approaches can be broadly classified into 2 main categories namely pre-known network features based approaches and pure sequence based approaches, which are briefly demonstrated in Fig 1. As shown by Fig 1, the former approaches rely on pre-known expression profile networks, interaction networks, protein 3D structures or gene co-expression networks for the determination of viral-host PPIs. For example, interolog mapping [19] based approaches map the known interacting and non-interacting viral-host protein pairs of source organism onto target organism to find most probable interacting viral-host protein sequence pairs on the basis of sequence similarity of corresponding interacting proteins. Domain-domain/motif interaction based approaches [20, 22, 23] usually leverage viral-host protein expression profiles, protein-domain profiles, domain-domain interactions, and motif-domain interaction information to determine potential interactions between viral and host proteins. Besides, few network based approaches make use of 3D structures of viral host proteins [21, 24] to identify interactive proteins on the basis of their proximity to experimentally identified 3D structures of viral-host protein or correspondence to structural homology models. Also, there exist few gene co-expression network based approaches which operate on the principle that genes with similar expression profiles are more likely to encode interacting viral-host proteins [25].

Critical analysis of pre-known network based viral-host PPI prediction approaches reveals that expression profiles based networks, motif-domain interaction networks are known for a very few organisms, and even these limited resources need periodic updates to match the volume and velocity of Proteomics data [19]. Furthermore, there is a possibility that the idea of selectively transferring the knowledge of viral-host PPIs from a rich domain to target domain may not prove fruitful for novel viral-host PPI prediction, because virus families do not carry sufficient intrinsic information such as sequence similarities among themselves in order to make transfer learning from other viruses possible. This reinforces the significance of purely sequence based predictive methodologies which exploit the relationships that exist between a series of amino acids within protein sequences to discriminate interactive viral-host proteins from non-interactive viral-host proteins.

To date, several purely raw sequence based computational approaches have been developed [12–17]. As machine or deep learning approaches require statistical representations of viral-host protein sequences to learn discriminative patterns which eventually help the model to perform viral-host PPI prediction. Hence, existing sequence features based computational approaches can be segregated into 2 stages: statistical representations generation and interaction prediction. Researchers have leveraged a variety of statistical representations generation (e.g repeat patterns and amino acid composition, residue physico-chemical properties, higher order residue frequency, unsupervised sequence embeddings) and computational interaction prediction approaches to effectively perform viral-host protein interactome analysis [13–16].

Cui et al. [17] transformed variable length viral-host protein sequence pairs into fixed length statistical vectors using the relative frequency of amino acids triplets and utilized support vector machine (SVM) classifier to infer viral-host PPIs. Abbasali et al. [16] reaped the benefits of 6 different feature encoders including pseudo amino acid composition (PAC), amino acid composition (ACC), network centrality computation metrics, tissue information, post-translational modification, and evolutionary information based features to encode viral-host protein sequences. Encoded feature vectors were passed to a meta-classifier based on the combination of Support vector machine (SVM), Naive Bayes (NB), Multi-Layer Perceptron (MLP), and Random Forest (RF). Fatma et al. [12] leveraged the PPI of various viruses and the amino acid representation scheme given by Cui et al. [17] to develop a robust SVM based methodology namely “DeNovo”.

Furthermore, Kim et al. [18] generated statistical representations of viral-host proteins using amino acid composition, relative occurrences of amino Acid triplets, and occurrence difference of amino Acid triplets that exist between human and virus proteins. They utilized SVM to discriminate interactive viral-host protein pairs from non-interactive viral-host protein pairs. Barnes et al. [15] presented “PIPE” which estimated the interaction of viral-host proteins by counting similar residues obtained by rotating multiple windows over viral-host protein sequences. Considering the direct relation of amount of amino acid repetitive patterns with interaction potential [26], Saud et al. [13] utilized the composition and repeats of amino acids for viral-host PPIs prediction.

In a nutshell, the prime focus of every new viral-host PPI predictor has been to learn optimal statistical representations of viral-host protein sequences. Among various statistical representations learning approaches, residue physicochemical properties based viral-host protein sequence representation learning schemes mark better performance than trivial residue frequency based approaches. However, these approaches neglect the relationships among residue segments as a function of context of entire viral-host protein sequences. In the marathon of generating the most effective representation of viral-host protein sequences, following the success of neural residue embeddings in a wide spectrum of Natural Language Processing [10] and Bioinformatics tasks [11], unsupervised embedding learning technique doc2vec [27] has shown better performance than repeat pattern and residue composition based sequence encoding, residue frequency based sequence encoding, and residue physicochemical properties based sequence encoding schemes. However, un-supervised embedding generation approaches primarily learn statistical representations of viral-host protein sequences by modeling only local context of residues, which entirely neglects long range dependencies and discriminative residue correlation important for accurate viral-host PPI prediction.

The paper in hand presents a unique way of generating rich statistical representations of viral-host protein sequences by fusing comprehensive local and global residue contextual information. In local residue context aware sequence encoding, we leverage physico-chemical properties to reduce viral-host protein sequences and categorize the residues present in reduced sequences into 6 different classes according to residue properties given by shannon entropy. statistical representations of viral-host protein sequences are generated through the assignment of residues to these respective classes. Local residue context aware encoding extracts short range dependencies of residues as well as residue semantic relatedness. Whereas, to capture long range dependencies and positional invariance of residues, in global residue context aware encoding, we compute composition and transition of viral-host protein sequences. Finally, to generate comprehensive statistical representations of viral-host protein sequences by capturing diverse types of information, we fuse the features extracted by local residue context aware encoding with the features extracted by global residue context aware encoding. Using optimized concatenated viral and host protein sequences representations, a robust machine learning framework LGCA-VHPPI based on deep forest model is developed which extracts correlation of features important to discriminate interactive viral-host protein pairs from non-interactive protein pairs, generalizeability of which does not affect much from complex data non-linearity, noise, and size of the training set.

To illustrate the effectiveness of proposed comprehensive local-global residue context aware viral-host protein sequence representation learning scheme based deep forest model, a detailed performance assessment is performed. Over 3 different benchmark viral-host PPI prediction datasets, local-global residue context aware viral-host protein sequence encoding scheme generate most comprehensive yet discrminative representation, outperforming only local residue context aware sequence encoding and global residue context aware sequence encoding respectively by a decent margin. Furthermore, optimized viral-host protein sequence representation assists the deep forest model to surpass the performance of various predictive approaches, outperforming the state-of-the-art doc2vec and Random Forest classifier based approach by 6%, 2%, and 2% in terms of matthews correlation coefficient over 3 different benchmark viral-host PPI prediction datasets.

## 2 Materials and methods

This section discusses proposed viral-host PPI prediction methodology, benchmark datasets and evaluation metrics used to assess the performance of proposed methodology.

### 2.1 Local-Global Residue Context Aware Sequence Encoding based Viral-Host PPI predictor (LGCA-VHPPI)

To determine the interactions among viral and host proteins, two different kinds of paradigms have been used in the literature to generate statistical representations of viral-host protein sequences [12, 14, 28]. One paradigm concatenates viral protein sequences with corresponding host protein sequences and then generates statistical representations of viral-host protein sequences. Whereas, the other paradigm generates statistical representations of viral protein and host protein sequences separately and concatenates both statistical representations before feeding them to a machine learning classifier. We utilize secondfirst paradigm to generate statistical representations of viral-host protein sequences using local and global residue context aware encoding (LGCAE) method. To illustrate better, consider a hypothetical viral protein sequence “ACGFXKLM” and host protein sequence “AKLJKMNOCPN”, using second paradigm and LGCAE method, we generate statistical representation of viral protein sequence (0.79, 0.89, 0.90,….) and host protein sequence (0.25, 0.49, 0.60,….) separately and then concatenate both statistical representations (0.79, 0.89, 0.90,….0.25, 0.49, 0.60,….) to formulate a single representation for viral-host protein sequence that is further fed to a deep forest classifier.

**Fig 2:**
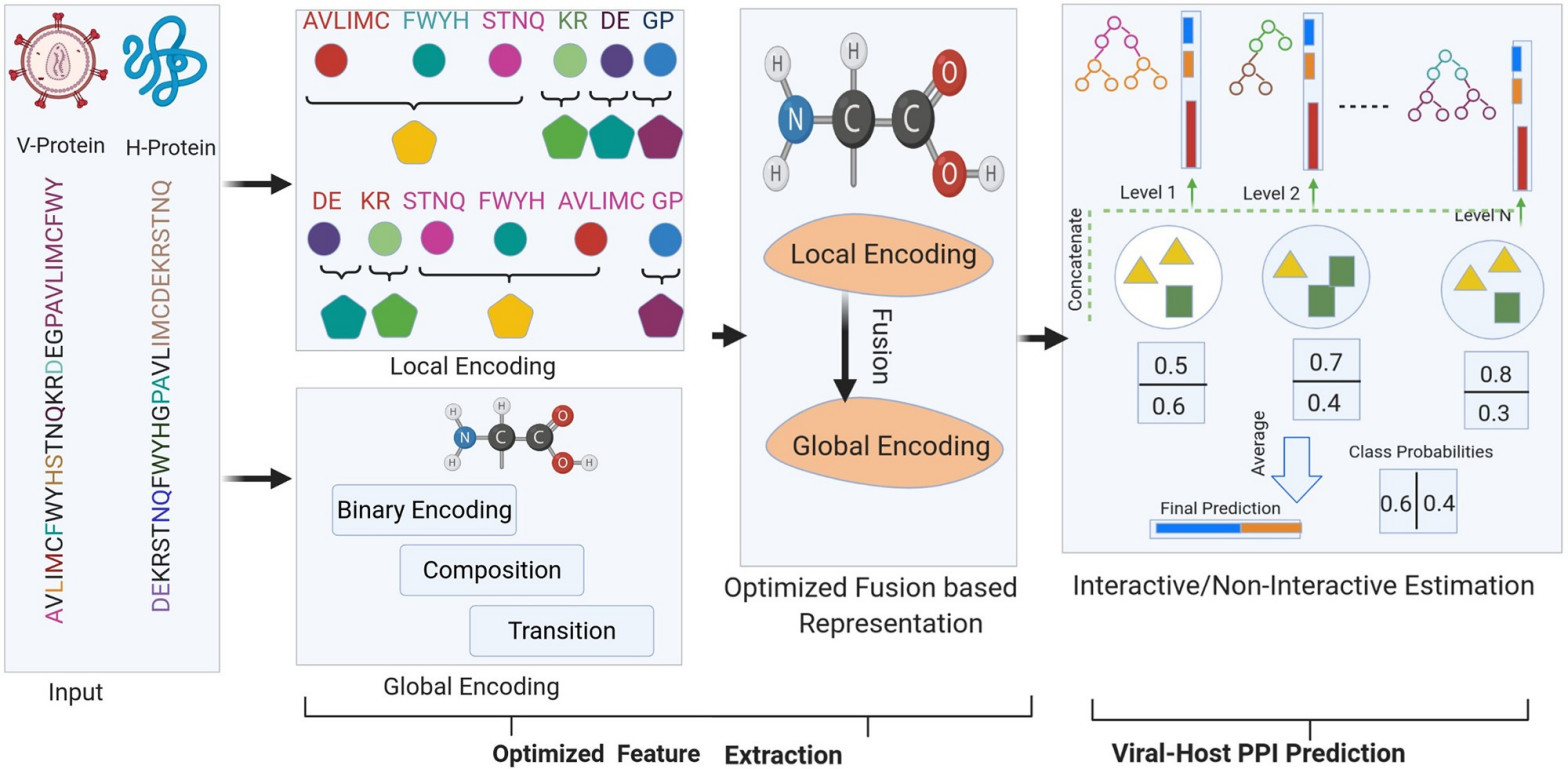
The workflow of proposed “LGCA-VHPPI” viral-host PPI predictor. First Block Represents Input Module, Second Block Shows the Paradigm of Local and Global Encoding, Third Block Integrates Local and Global Encodings, and Last Block Reveals the Probabilistic Paradigm of Deep Forest.

The workflow of proposed LGCA-VHPPI predictor is demonstrated in Fig 2. LGCA-VHPPI mainly consists on 2 core modules: local and global residue context aware encoding generation of viral-host protein sequences and viral-host PPI prediction using deep forest model, details of both modules is provided in following sub-sections.

#### 2.1.1 Local-global residue context aware encoding generation of viral-host protein sequences

Proposed Local-Global Residue Context Aware Encoding (LGCAE) scheme is the fusion of 2 different modules 1) Local Residue Context Aware Sequence Encoding (LCAE) and 2) Global Residue Context Aware Sequence Encoding (GCAE), paradigms of which are explained in following sub-sections.

*Local residue context aware encoding generation of viral-host protein sequences*. Using the collection of viral-host protein sequences, local residue context aware encoder “LCAE” computes a dictionary D = {A,R,N,D,C,E,Q,G,H,I,L,K,M,F,P,S,T,W,Y,V} containing 20 unique amino acids. We reduce the distribution of 20 unique amino acids in the viral-host protein sequences to 6 unique classes of amino acids using physico-chemical properties of amino acids such as polarity and hydrophobicity. Considering Shannon entropy of amino acids properties [29], rather than treating every amino acid as a unique symbol in entropy computation, it categorizes the amino acids into 6 different classes namely aliphatic (AVLIMC), polar (STNQ), positive (KR), negative (DE), aromatic (FWYH), and special (G,P) based on especial conformational properties [30]), which are provided in Table 1. With an aim to better compute the distribution of 6 different classes based on unique amino acids, iteratively, from all 6 classes, 3 amino acids classes are treated as one group whereas other classes are treated as individual groups. In this way, from six unique amino acid classes, we obtain 20 different patterns shown in Table 2 where each pattern is comprised of four unique letters B, J, O, and U. We use all 20 different patterns to compute effective statistical representations of viral-host protein sequences.

**Table 1:** Amino acid (Residue) categorization into 6 different classes.

*Descriptor*	*Property*	*Categorization*
A1	Aliphatic amino acid	A,V,L,I,M,C
A2	Aromatic amino acid	F,W,Y,H
A3	Polar amino acid	S,T,N,Q
A4	Positive amino acid	K,R
A5	Negative amino acid	D,E
A6	Special conformations	G,P

**Table 2:** Twenty possible groups representing different combined patterns of residues which are described using 4 letters B, J, O, and U.

V	B	J	O	U
v1	{A1, A2, A3}	A4	A5	A6
v2	{A1, A2, A4}	A3	A5	A6
v3	{A1, A2, A5}	A3	A4	A6
v4	{A1, A2, A6}	A3	A4	A5
v5	{A1, A3, A4}	A2	A5	A6
v6	{A1, A3, A5}	A2	A4	A6
v7	{A1, A3, A6}	A2	A4	A5
v8	{A1, A4, A5}	A2	A3	A6
v9	{A1, A4, A6}	A2	A3	A5
v10	{A1, A5, A6}	A2	A3	A4
v11	{A2, A3, A4}	A1	A5	A6
v12	{A2, A3, A5}	A1	A4	A6
v13	{A2, A3, A6}	A1	A4	A5
v14	{A2, A4, A5}	A1	A3	A6
v15	{A2, A4, A6}	A1	A3	A5
v16	{A2, A5, A6}	A1	A3	A4
v17	{A3, A4, A5}	A1	A2	A6
v18	{A3, A4, A6}	A1	A2	A5
v19	{A3, A5, A6}	A1	A2	A4
v20	{A4, A5, A6}	A1	A2	A3

Furthermore, to better describe the process to generate statistical representations of viral-host protein sequences using 20 different patterns, Let us consider a viral host protein sequence *S* = *S*_1_…‥*S*_*n*_ of length *n* where *S*_*q*_ represents a particular residue, an initial level numerical representation is generated by iteratively mapping the sequence *S* to every group *v*_*i*_ expressed in terms of 4 unique letters (B, J, O, U) and replacing 4 unique letters with corresponding ASCII values. To optimize initial level numerical representation of viral-host protein sequences generated for each group *v*_*i*_, following the success of transformer based approaches to capture positional information of residues for diverse Natural Language Processing and Bioinformatics tasks [31, 32], we capture positional information of 4 unique letters to better track short range dependencies of residues. For all 4 letters, we compute their total occurrences in given viral-host protein sequence *S* represented as *B*_*n*_, *O*_*n*_,*J*_*n*_, *U*_*n*_ as well their count in initial j entries (*B*_*j*_, *O*_*j*_,*J*_*j*_, *U*_*j*_) of sequence where j is iteratively updated until it matches the length *n* of sequence. Using total occurrences of 4 unique letters (*B*_*n*_, *O*_*n*_,*J*_*n*_, *U*_*n*_) and their iteratively increasing counts ((*B*_*j*_, *O*_*j*_,*J*_*j*_, *U*_*j*_)), we capture alteration in positional bits of all 4 unique letters in such a manner that odd and even position values fall in range of cosine and sine functions, mathematical expressions of which are given in Eqs 1 and 2 respectively.

$$XS_{q}(v_{i})=\{\begin{array}{l} \text{cos}\;\frac{\pi }{2}+\frac{\pi }{2}\frac{B_{j}}{B_{n}+1}\;if\;S_{q}=B\\ \text{cos}\;\frac{\pi }{2}+\frac{\pi }{2}\frac{J_{j}}{J_{n}+1}\;if\;S_{q}=O\\ \text{cos}\;\pi +\frac{\pi }{2}\frac{O_{j}}{O_{n}+1},\;if\;S_{q}=J\\ \text{cos}\;\frac{3\pi }{2}+\frac{3\pi }{2}\frac{U_{j}}{U_{n}+1}\;if\;S_{q}=U \end{array}$$ (1)

$$YS_{q}(v_{i})=\{\begin{array}{l} \text{sin}\frac{\pi }{2}+\frac{\pi }{2}\frac{B_{j}}{B_{n}+1}\;if\;S_{q}=B\\ \text{sin}\frac{\pi }{2}+\frac{\pi }{2}\frac{J_{j}}{J_{n}+1}\;if\;S_{q}=O\\ \text{sin}\pi +\frac{\pi }{2}\frac{O_{j}}{O_{n}+1}\;if\;S_{q}=J\\ \text{sin}\frac{\pi }{2}+\frac{\pi }{2}\frac{U_{j}}{U_{n}+1}\;if\;S_{q}=U \end{array}$$ (2)

Using *XS*_*q*_(*v*_*i*_) and *YS*_*q*_(*v*_*i*_) obtained from Eqs 1 and 2, 4 different normalized vectors can be computed for each group using mathematical expression given in Eqs 3 and 4 respectively.

$$\begin{array}{c} \left[\begin{array}{cc} XN\left(v_{i}\right)=\frac{1}{len\left(seq\right)\times \sum XS_{q}\left(v_{i}\right)} & \\ YN\left(v_{i}\right)=\frac{1}{len\left(seq\right)\times \sum YS_{q}\left(v_{i}\right)} \end{array}\right] \end{array}$$ (3)

In Eqs 3 and 4, different mathematical formulas compute floating point values where in each mathematical formula, len(seq) denotes the number of residues present in the sequence, and ∑*XS*_*q*_(*v*_*i*_), ∑*YS*_*q*_(*v*_*i*_), ∑*A*(*v*_*i*_), and ∑*B*(*v*_*i*_) denote the sum of floating point values present in respective collections (*XS*_*q*_(*v*_*i*_), *YS*_*q*_(*v*_*i*_), *A*(*v*_*i*_), and *B*(*v*_*i*_)).

$$\begin{array}{c} \left[\begin{array}{cc} A\left(v_{i}\right)={\left(XS_{q}\left(v_{i}\right)-XN\left(v_{i}\right)\right)}^{2} & \\ B\left(v_{i}\right)={\left(YS_{q}\left(v_{i}\right)-YN\left(v_{i}\right)\right)}^{2} & \\ AXN\left(v_{i}\right)=\frac{1}{len\left(seq\right)-1\times \sum A\left(v_{i}\right)} & \\ BXN\left(v_{i}\right)=\frac{1}{len\left(seq\right)-1\times \sum B\left(v_{i}\right)} & \end{array}\right] \end{array}$$ (4)

Then, LCAE vector with respect to each group can be computed by concatenating 4 normalized vectors (Eq 5).

$$\begin{array}{c} LCAE_{G1}=XN\left(v_{i}\right)⊕YN\left(v_{i}\right)⊕AXN\left(v_{i}\right)⊕BXN\left(v_{i}\right) \end{array}$$ (5)

$$\left[LCAE=LCAE_{G1}⊕LCAE_{G2}⊕,\ldots LCAE_{G10}\right]$$ (6)

As we have 20 different patterns, so comprehensive semantic relatedness and short range residue dependencies aware (LCAE) viral-host protein sequences representations can be generated by combining the representation of 20 different patterns (Eq 6). For each viral-host protein sequence, considering 4 normalization factors for every one out of 20 patterns, LCAE generates a 80-dimensional 40-dimensional vector (20 × 4) (Eq 6).

*Global residue context aware encoding generation of viral-host protein sequences*. Global residue context aware encoding (GCAE) captures comprehensive composition and transition information of residues. First, using the collection of viral and host protein sequences, a dictionary D = A,R,N,D,C,E,Q,G, H,I,L,K,M,F,P,S,T,W,Y,V containing 20 unique amino acids is computed. Then for a given viral or host protein sequence S = S1,….,Sn; of length *n* where *Si* represents a particular amino acid present in dictionary D, GCAE generates a sparse 20×*n* matrix *A* where 20 unique amino acids act as row indices and sequence residues *Sn* act as column indices. Sparse matrix *A* looks like:

$$\begin{array}{c} A=[\begin{array}{ccccc} & S_{1} & S_{2} & ... & S_{n}\\ A & a_{11} & a_{12} & ... & a_{1n}\\ R & a_{21} & a_{22} & ... & a_{2n}\\ \vdots & \vdots & \vdots & \vdots & \vdots \\ V & a_{20,1} & a_{20,2} & ... & a_{20,n} \end{array}]=a_{\left(i,j\right)}=\{\begin{array}{c} 1,if\;D(i)=S(j)\\ 0,others \end{array}\} \end{array}$$ (7)

Sparse matrix *A* is distributed with the values of 0 and 1 as every cell represented as *a*_(*i*,*j*)_ gets the value of 1 if amino acid present in row index D(i) matches with amino acid present in column index S(j) and 0 otherwise. As basic working principal of global context aware encoder is to capture composition and transition of amino acids in viral-host protein sequences. Here in the matrix, length of each amino acid is equal to the length of particular viral or host protein sequence. Hence, instead of finding the composition and transition of amino acid by taking the full rows of the matrix, we divide the rows into *L* number of sub-rows/sub-regions. Depending upon the size of input viral or host protein sequence and value of *L*, it is highly likely that one of many sub-regions is shorter in length due to low leftover amino acids in the input sequence. For example, for a sequence containing 25 amino acids, if we select L = 2, then two sub-regions will have the different number of amino acids such as 13, and 12 respectively. However, GCAE operates on same size sub-regions. Hence, to generate same size sub-regions, pre-processing strategy will first add one non-amino acid letter B at the end of input sequence and then divides the sequence into 2 sub-regions where each sub-region will have 13 amino acids. In other words, for a given viral or host protein sequence, before the generation of *L* number of sub-regions, pre-processing strategy extends the length of given sequence using the copy padding trick where it adds a non amino-acid letter (e.g B) at the end of input sequence as many times as it makes the generation of same size L number of sub-regions possible. After generating same size sub-regions, we compute composition and transition with respect to each sub-region.

The composition computes the frequency of ‘1”, ‘11”, and ‘111” in each row of sparse matrix to capture the proportion of various residues within sequence and their most dominant contexts.

$$\begin{array}{c} Composition=Frequency\;of\;1\;⊕Frequency\;of\;11\;⊕\;Frequency\;of\;111 \end{array}$$ (8)

As composition computes frequencies of three different trends for each sub-vector ∈ L, therefore dimensions of composition vector can be computed as:

$$\begin{array}{c} Composition\;Vector=20\times L\times 3 \end{array}$$ (9)

Whereas, transition computes the frequency of ‘1” followed by ‘0” and ‘0” followed by ‘1” in each row of the sparse matrix to capture the dominant change in the context of the residue.

$$\begin{array}{c} Transition=Frequency\;of\;1-0\;⊕Frequency\;of\;0-1 \end{array}$$ (10)

Because, transition computes two different trends for each sub-vector ∈ L,, hence dimensions of transition vector can be computed as:

$$\begin{array}{c} TransitionVector=20\times L\times 2 \end{array}$$ (11)

$$\begin{array}{c} GCAE\;Vector=Composition\;Vector\;⊕\;Transition\;Vector \end{array}$$ (12)

**Fig 3:**
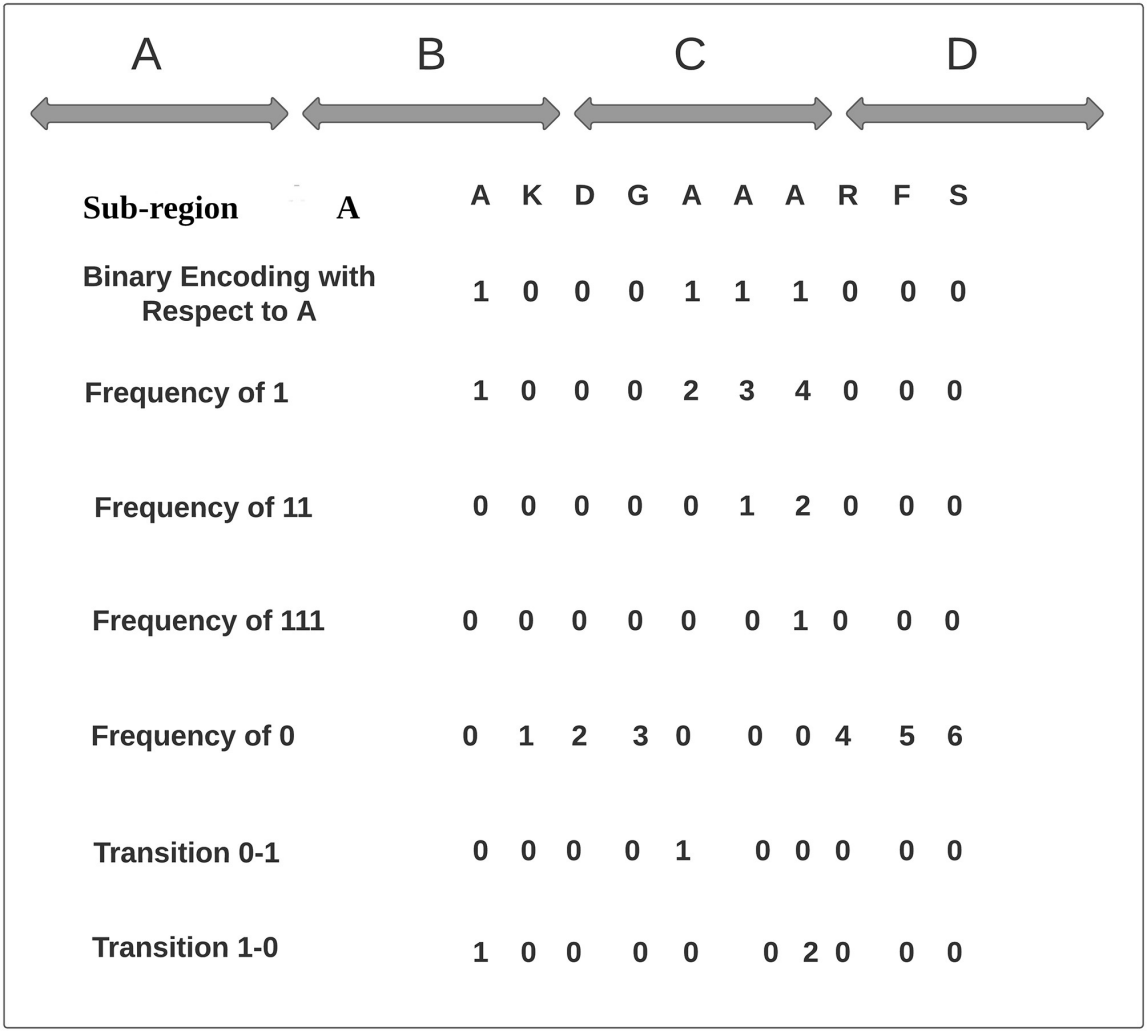
Segregation of a hypothetical sequence of 40 amino acids into 4 sub-regions of 10 amino acids using L = 4 which are represented as A, B, C, D. Computation of Global Residue Context Aware Encoding for One Amino Acid A in First Sub-Region A is Shown.

By concatenating composition and transition vectors, GCAE viral-host protein sequence vector is generated. To better illustrate the working paradigm of global residue context aware encoding, a hypothetical example is demonstrated in Fig 3.

Consider a hypothetical viral protein sequence containing 40 residues. To generate statistical representations of this viral protein sequence, GCAE first generates a 2-dimensional matrix of size amino acid: 20 ×size of sequence (40). In this matrix, 20 amino acids act as rows indices and 40 amino acids present in sequence as column indices and each cell of every row gets the value of 1 or 0 depending on the match of row index with column index. This sequence is divided into four sub-regions of 10 amino acids by taking sub-region size L = 4 where GCAE computes composition and transition of residues with respect to every sub-regions across entire rows of sparse matrix. To better illustrate the working paradigm of GCAE, Fig 3 describes the process of learning representation for amino acid “A” using one of the 4 sub-regions. As shown by the Fig 3, from 2-dimensional sparse matrix, for amino acid A, binary encoding of sub-region “AKDGAAARFS” looks like ‘1000111000”. Using binary representation, GCAE computes composition by counting the frequency of ‘1”, ‘11”, and ‘111” whereas transition computes the frequency of 1 followed by 0 and 0 followed by 1 patterns. Statistical analysis indicates that this sub-region has four ‘1”, six ‘0”, two “11”, one “111”, one 0–1 transition, and two 1–0 transitions. As there are four sub-regions (L = 4) so this sub-region will get a 400 dimensional feature vector (Amino Acid: 20 ×L:4 ×Composition:3 + Amino Acid: 20 ×L:4 ×Transition:2 = 400 dimensional vector).

*Fusion of local and global residue context aware sequence encoders*. Local-Global context aware sequence encoding is generated by fusing local sequence vector produced by LCAE with global sequence vector produced by GCAELocal-Global context aware sequence encoding is generated by fusing 40-dimensional local sequence vector with 400 dimensional global sequence vector which ensures that statistical representations of viral or host protein sequences contain comprehensive short and long range positional as well as semantic information of residues important to discriminate interactive viral-host protein sequence pairs from non-interactive viral-host protein sequence pairs.

#### 2.1.2 Viral-host PPI prediction using deep forest

To distinguish interactive viral-host protein sequences from non-interactive ones, we use a deep forest model. Deep forest model comprises of a sequence of random forests where each random forest is trained over sequences statistical vectors440-dimensional sequence vectors produced by proposed LGCAE scheme and yields a 2-dimensional probability class vector. Contrary to deep neural networks, deep forest model is extremely effective in learning hyper-level representation with lowest cost and has shown great promise in multifarious bioinformatics tasks [33]. Deep forest classifier takes the interactive and non-interactive class probability scores of all individual trees into account to predict more stable and accurate probability scores for both classes across different datasets.

Furthermore, a number of hyperparameters can be tuned in a deep forest model such as decision trees estimators, maximum depth of individual decision tree estimator, node splitting criteria, minimum samples used to split on at the internal node of decision tree estimators, amount of random features used at every node for splitting, maximum leaf nodes, and size of bootstrapped dataset used to train every decision tree [34, 35]. Pre-dominant studies consider number of decision tree estimators, maximum depth of decision trees, and splitting criteria to be the most influential hyperparameters of a deep forest model [34, 35]. Building on this, deep forest model is optimized using the automated parameters search paradigm namely Grid Search [36] where initial estimator range is varied from 200-to-400, maximum depth from 500-to-800 [34, 35], and splitting criteria from gini, entropy, to logloss. On Barman dataset [28], deep forest has achieved best performance with gini splitting criteria, 291 estimators, and maximum depth of 702. On Denovo [12] and Yang et al. [14] datasets, deep forest has achieved best performance with gini splitting criteria, 300 estimators, and default maximum depth.

### 2.2 Benchmark viral-host protein-protein interaction prediction datasets

**Fig 4:**
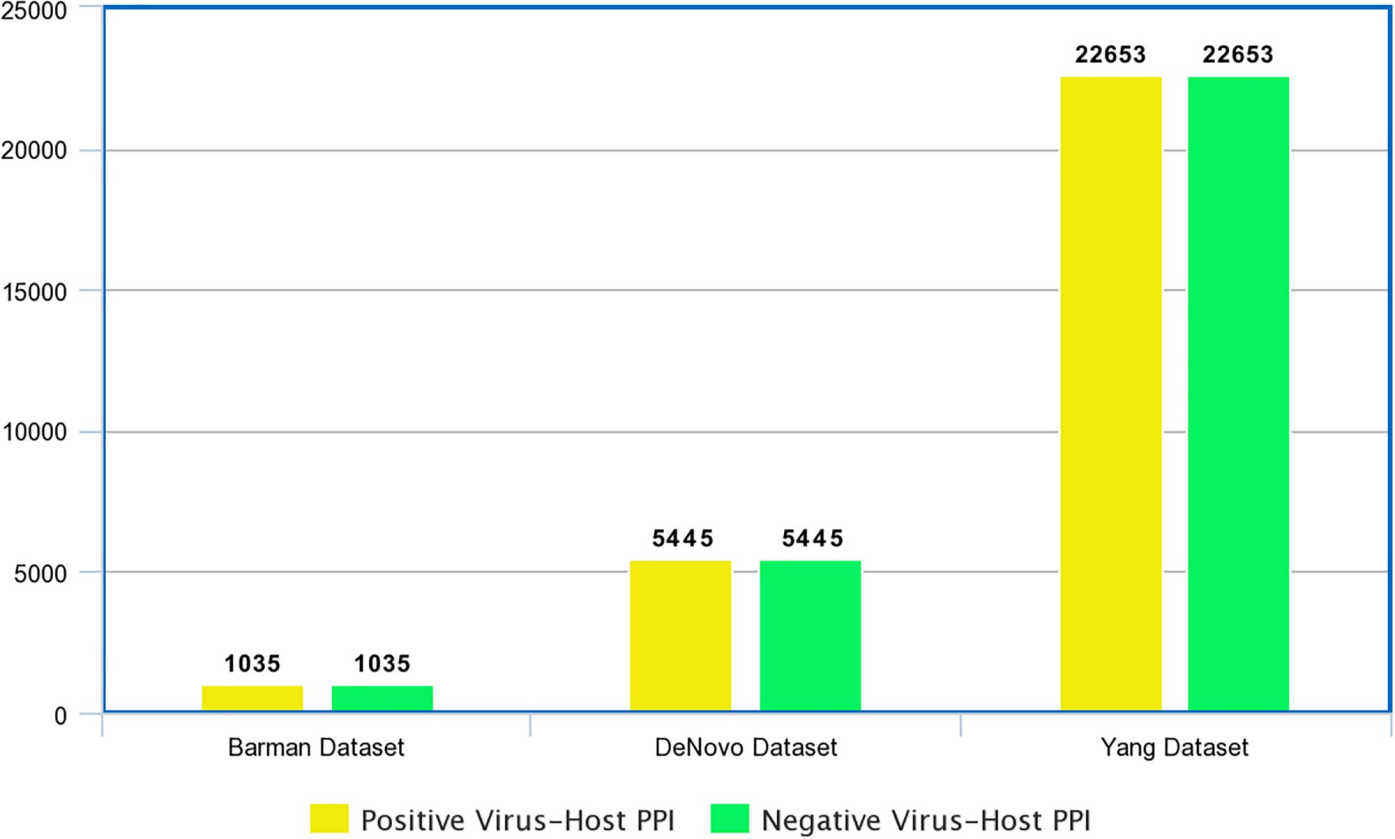
Statistics of 3 different benchmark viral-host PPI prediction datasets.

In order to evaluate the integrity of proposed viral-host PPI predictor “LGCA-VHPPI”, we have collected 3 different benchmark datasets from 3 different research studies [12, 14, 28], statistics of which are provided in Fig 4.

Barman et al. [28] prepared a viral-host PPI dataset using a virus-protein interaction repository VirusMINT [37]. VirusMINT contains viral-host PPIs of different viruses including papilloma virus, human immunodeficiency virus, hepatitis-B, and hepatitis-C virus (HCV) [37]. Considering the inclusion of quite similar sequences introduces undesirable biases especially during the extraction of average values or pre-dominant trends which lead to model over-fitting. Authors found a total of 2,707 interactions from VirusMINT [37] database. They eliminated 1,224 repetitive interactions (viral protein A-host protein B and host protein B-viral protein A). Furthermore, using a public resource namely InterPro [38] that facilitates functional analysis of the proteins by categorizing them into various families and inferring their domains, authors discarded 337 interacting protein pairs for which “InterPro” [38] had no domain information. From remaining 1,146 interactions, they found 1,035 interactions between viral and human proteins which were used to formulate a positive dataset. To generate a negative dataset, using positive-to-negative ratio of 1:1, they arbitrarily selected 1,035 viral-host protein pairs as negative which did not appear in positive set.

Another benchmark viral-host PPI dataset (DeNovo dataset) was prepared by Fatma et al. [12] using VirusMentha database [39]. They collected 453 viral proteins belonging to 173 different viral species. Using VirusMentha database [39], they extracted 2,357 human proteins and 453 viral proteins found a total of 5,753 PPIs between viral and human proteins. In-complete and duplicate viral-host PPI were eliminated to acquire a positive dataset of 5,445 viral-host PPIs between 445 viral proteins and 2,340 human proteins. Like Barman et al. [28], they prepared a negative dataset of 5,445 viral-host PPIs using random pairing of viral and human proteins which did not appear in positive set.

Third benchmark viral-host PPI dataset was prepared by Yang et al. [14]. They utilized Host Pathogen Interaction Database [40] which contained manually formulated virus-host PPIs as well as molecular interactions of other public PPI databases. To obtain a quality virus-host PPI dataset, PPIs identified by MS large-scale experiments, redundant PPIs, non-physical PPIs, and PPI sequences having less than 30 amino acids or greater than 5000 amino acids were eliminated. In this manner, they obtained 22,653 interactive virus-host PPIs. To generate negative samples, they leveraged dissimilarity based negative sampling approach [12]. More specifically, according to dissimilarity based negative sampling approach, if two viral proteins X and Y have the sequence similarity of greater than 0.3 [41] and X interacts with host protein C, then protein pair Y-C can not be considered as a negative sample. Using positive-to-negative ratio of 1:10, authors arbitrarily selected viral protein from the collection of interactive samples and host proteins from SwissProt [42] repository to create a negative dataset.

### 2.3 Evaluation criterion

Following the evaluation criteria of different researchers used to evaluate their proposed viral-host PPI prediction methodologies, we evaluate the performance and generalizeability of proposed viral-host PPI prediction methodology LGCA-VHPPI in terms of 8 different evaluation metrics [14], mathematical expressions of which are given below:

$$f(x)=\{\begin{array}{l} \text{Accuracy}\;\text{(ACC)}=\frac{\left(O_{-}^{+}+(O_{+}^{-}\right)}{\left(O^{+}+O^{-}\right)}\\ \text{Specificity}\;\text{(SP)}=(O_{+}^{-}/(O_{+}^{-}+F_{-}^{+}))\\ \text{Precision}\;\text{(PR)}=\frac{\left(O_{-}^{+}\right)}{\left(O^{+}\right)}\\ \text{Sensitivity}\;\text{(SN)}=\frac{\left(O_{-}^{+}\right)}{\left(O_{-}^{+})+(F_{+}^{-}\right)}\\ \text{MCC}=\frac{\left((O_{-}^{+}\times O_{+}^{-})-(F_{-}^{+}\times F_{+}^{-})\right)}{\left(O_{-}^{+}+F_{-}^{+})\times (O_{-}^{+}+F_{+}^{-})\times (O_{+}^{-}+F_{-}^{+})\times (O_{+}^{-}+F_{+}^{-}\right)}\\ \text{F1-score}=2\times \frac{\left[PR\times SN\right]}{\left[PR+SN\right]} \end{array}$$ (13)

Here in Eq 13, O^+^ refers to the count of false and true positives, O^-^ represents the count of false and true negatives, true positives are represented by $O_{-}^{+}$ and true negatives are represented as $O_{+}^{-}$. Whereas, false positives and false negatives are represented as $F_{-}^{+}$ and $F_{+}^{-}$ respectively. Accuracy (ACC) estimates how many interactive as well as non-interactive viral-host protein sequence pairs are correctly predicted out of total predictions made by the model. Sensitivity and specificity estimate the model capability to accurately identify interactive viral-host protein sequence pairs and non-interactive viral-host protein sequence pairs respectively. Whereas, precision estimates the proportion of interactive viral-host protein sequence pairs from the model predictions. Taking the orthogonal relationship of precision and sensitivity into account, F1-score produces a more balanced estimate of model performance by computing a harmonic mean between precision and sensitivity. In order to ensure that the performance of a machine learning model is not biased towards the size of certain class, 3 evaluation measures including matthews correlation coefficient (MCC), area-under receiver operating charateristics, and area-under precision recall curve are used. While MCC takes all four confusion matrix categories such as true positive, true negative, false positive, false negative into account as well size of interactive and non-interactive class to estimate model performance. AU-ROC and AU-PRC compute model performance at different thresholds by comparing actual classes with predicted probabilities. AU-ROC helps to visualize the trade-off among true positive rate and false positive rate produced by the model by equivalently caring true negatives and true positives. On the other hand, prime focus of AU-PRC is to visualize the trade-off between positive predicted value and true positive rate, focusing on positive class and paying less attention to negative class in order to effectively reveal model capability of determining interactive viral-host protein sequence pairs.

## 3 Experimental setup

The proposed local-global residue context aware viral-host protein sequence representation learning scheme and deep forest are implemented using python based open source libraries (e.g scipy, scikit-learn).

To perform a fair performance comparison of proposed LGCA-VHPPI predictor with existing predictors, following Barman et al. work [28], we have performed 5-fold cross validation for Barman Dataset [28]. For DeNovo dataset, a standard train test split is available, therefore, we have utilized the same standard split to evaluate LGCA-VHPPI approach. For third benchmark dataset [14], authors have provided 3 different train test splits, hence, following the work of Yang et al. [14], we evaluate LGCA-VHPPI on 3 distinct data splits and compute the mean performance values.

## 4 Results and discussion

This section illustrates the performance of global context aware encoder with different number of sub-regions. It illustrates the performance values and generalizability achieved by deep forest classifier under the hood of three different viral-host protein sequence representation learning schemes solely based on the local distribution of residues, global distribution of residues, and fused local-global distribution of residues using three different benchmark datasets. Furthermore, it performs a fair performance comparison of proposed local-global residue context aware sequence encoding and deep forest based methodology LGCA-VHPPI with existing viral-host PPI predictors namely Barman et al. [28] approach, Alguwzizani et al. [13] approach, Fatma et al. [12] approach, and state-of-the-art Yang et al. [14] approach using benchmark Barman [28], Denovo [12] and Yang et al. datasets [14] in terms of 7 different evaluation measures.

### 4.1 Optimization of parameter L related to different number of sub-regions in global context aware encoding approach

**Fig 5:**
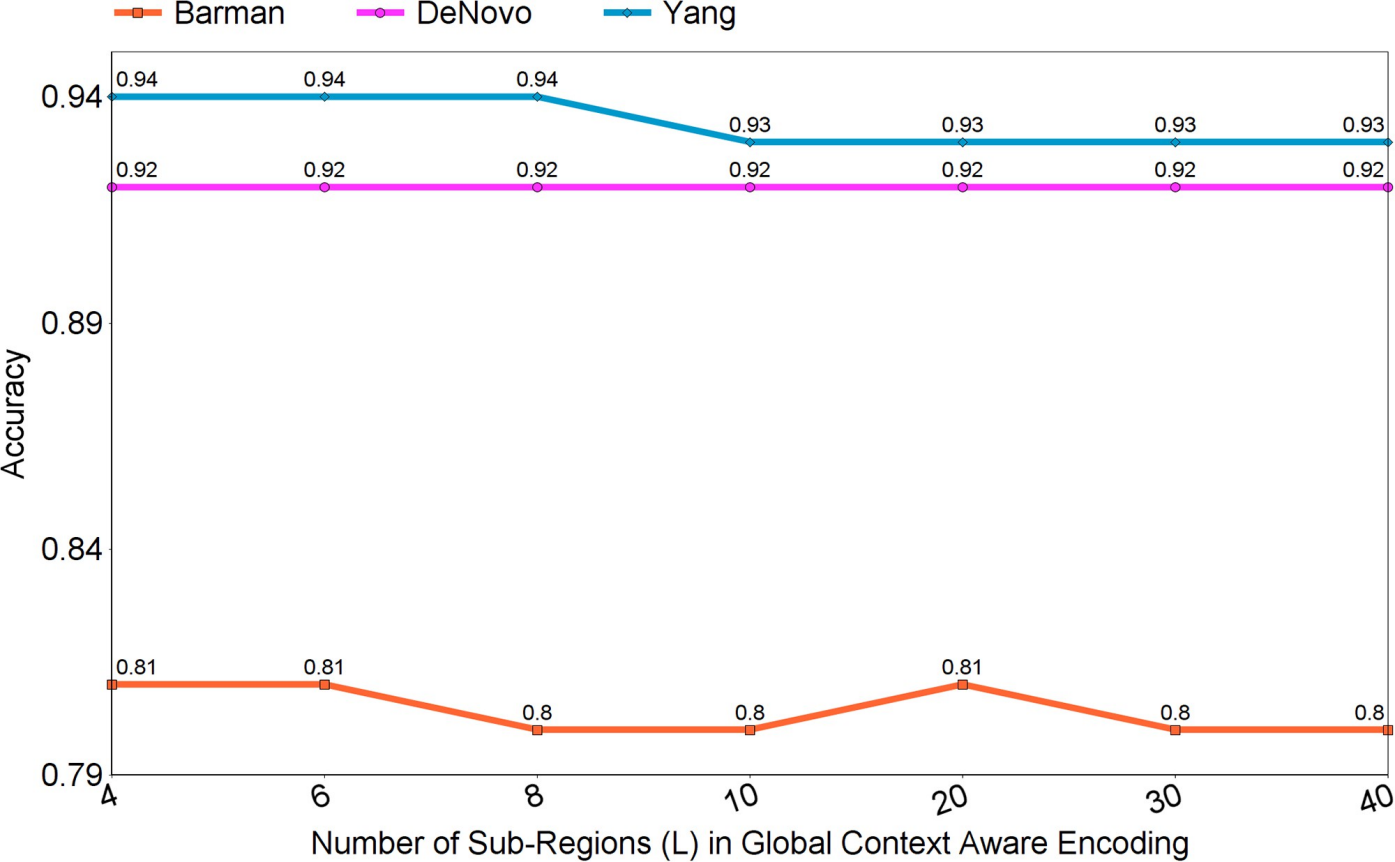
Impact of different size (L) sub-regions on the performance of global context aware encoding across three benchmark datasets namely Barman [28] and Denovo [12], and Yang et al dataset [14].

To find the optimal value of parameter *L* related to number of sub-regions in global context aware encoding (GCAE) approach, we take 80% sequences of Barman dataset [28], and complete training set of Denovo [12] and Yang et al. [14] datasets. On all three datasets, we tweak the parameter *L* from 4 to 40 to assess the impact of different number of sub-regions on the performance of GCAE produced using deep forest classifier for the task of viral-host PPI prediction. As shown by the Fig 5, on Barman dataset, GCAE performance only slightly fluctuates with different number of sub-regions, on Denovo dataset, GCAE performance remains same across different number of sub-regions (*L*), and on Yang et al dataset, GCAE performance remains at 94% until 8 sub-regions and drops by only 1% afterwards. This indicates that parameter *L* related to different number of sub-regions does not significantly influence the performance of GCAE across all three benchmark datasets, hence, we have divided viral and host protein sequences in 6 sub-regions to generate GCAE based representations across all three datasets.

### 4.2 Performance assessment of deep forest using different statistical representations learning schemes over 3 different benchmark viral-host PPI prediction datasets

**Fig 6:**
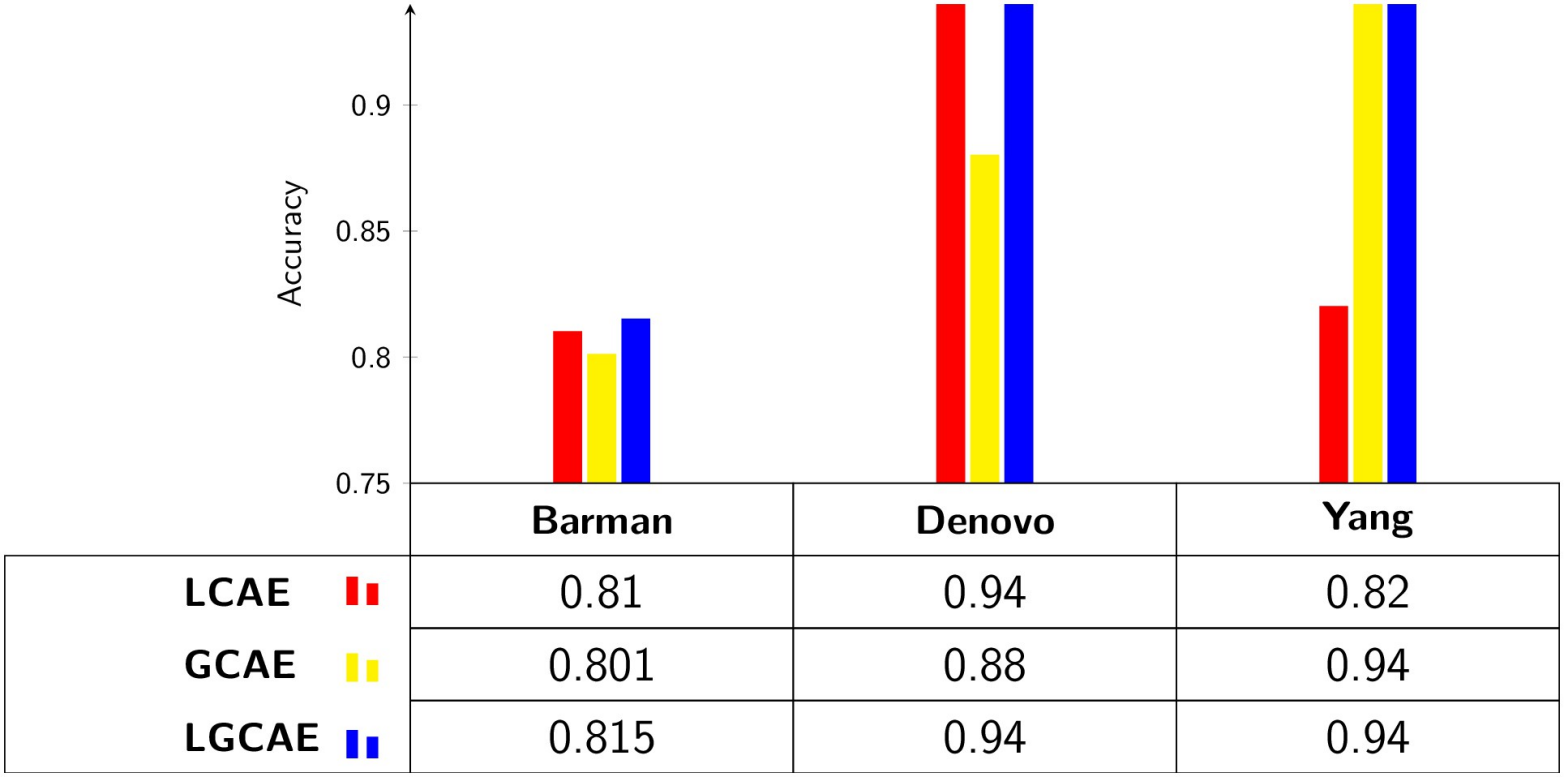
Performance comparison of local, global, and local-global residue context aware viral-host protein sequence representation learning schemes using deep forest model over 3 benchmark viral-host PPI prediction datasets.

Fig 6 illustrates the performance values produced by deep forest model across 3 benchmark datasets by utilizing 3 different statistical representations learning schemes to analyze which representation learning scheme dominantly assists deep forest model to extract discriminative patterns for accurate viral-host PPI prediction. Performance analysis in terms of accuracy indicates that feeding deep forest model with global residue context aware based sequence encoding (GCAE) marks the lowest performance on two of the benchmark datasets. This is primarily due to the fact that GCAE mainly focuses on composition and transition, long-range dependencies, and positional invariance of residues and neglects local contextual information of residues while generating viral-host protein sequence encoding. In contrast, local residue context aware based sequence encoding (LCAE) marks better performance than GCAE across most benchmark datasets as it captures semantic relatedness of residues, short range dependencies of residues by transforming the sequence into sub-sequences and sub-sequences into unique groups. On average, LCAE raises GCAE performance by 2%. Among all 3 sequence encoding schemes, proposed local-global residue context aware based sequence encoding (LGCAE) achieves the best performance across all 3 benchmark datasets mainly due to the integration of comprehensive local and global residue contextual information and ability to model precise and long discriminative residue combination distribution important for viral-host PPI prediction. In 3 different benchmark datasets, proposed LGCAE raises LCAE average performance by 4% and GCAE average performance by 3%.

**Fig 7:**
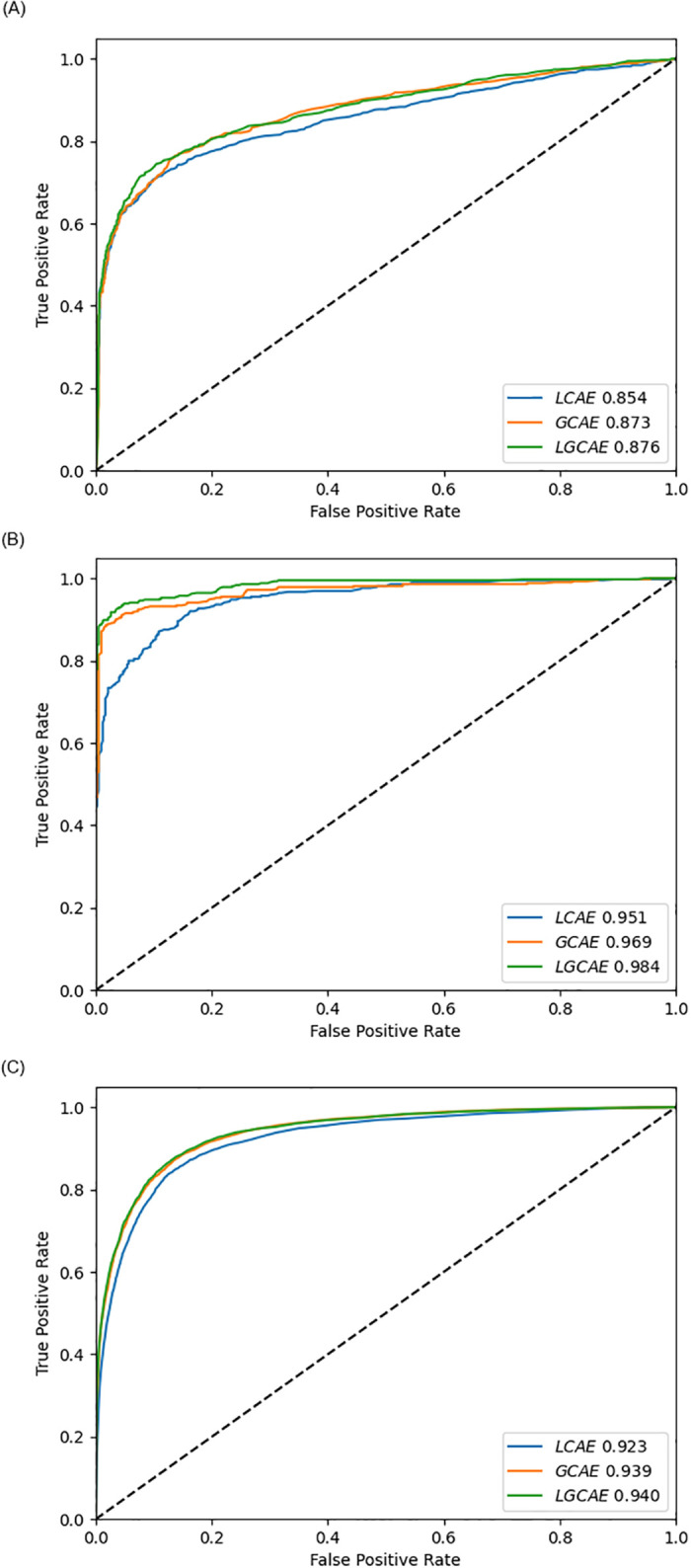
AU-ROC performance figures produced by proposed LGCA-VHPPI on 3 benchmark viral-host PPIs datasets. (a) Barman et al dataset. (b) DeNovo et al dataset. (c) Yang et al dataset.

**Fig 8:**
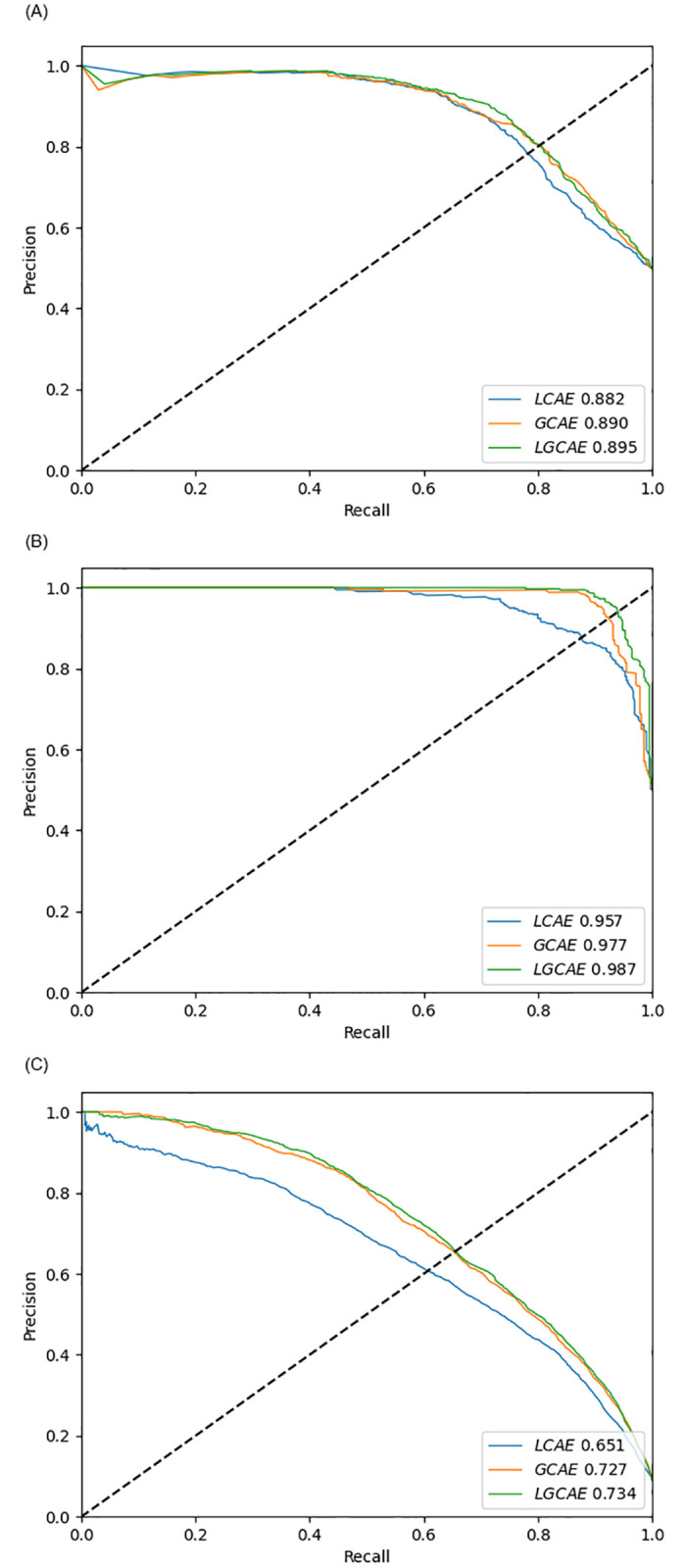
AU-PRC performance figures produced by proposed LGCA-VHPPI on 3 benchmark viral-host PPIs datasets. (a) Barman et al dataset. (b) DeNovo et al dataset. (c) Yang et al dataset.

Furthermore, to reveal the true generalization potential of deep forest across 3 different benchmark datasets under the hood of 3 statistical representations learning schemes, performance of LCAE, GCAE, and LCGAE is compared using AU-ROC and AU-PRC, shown in Figs 7 and 8 respectively. In Fig 7, AU-ROC scores produced by 3 different statistical representations learning schemes indicates that, just like accuracy, once again LCGAE achieves better degree of separability followed by GCAE and LCAE across all 3 datasets. LGCAE raises GCAE degree of separability by the average figure of 1% and LCAE degree of separability by the average figure of 3%, achieving the maximum AU-ROC score of 98% on Denovo dataset [12]. Similarly, analyzing the deep forest performance produced using 3 distinct statistical representations learning schemes across 3 different benchmark datasets in terms of AU-PRC reveals that LGCAE is neither biased towards type I error nor type II error. On average, LGCAE raises AU-PRC score of GCAE by 1% and LCAE by 4%, achieving the peak AU-PRC score of 99% on Denovo dataset [12].

In a nutshell, the main reason for LGCAE better performance and generalizeability is the context coverage which is highest in LGCAE as compared to LCAE and GCAE. LGCAE fuses different contextual granularity of sequence residues to capture generic yet discriminative distribution of residues which leads to better generalizeability across different viral species by ensuring deep forest model does not over-specialize certain regions of viral-host protein sequences. Furthermore, despite very different sequence to label distribution, proposed LGCAE based deep forest approach manages to produce promising accuracy, AU-ROC and AU-PRC across all 3 different benchmark datasets, revealing that the performance of proposed approach is not influenced much by the size of training data as well as size of positive or negative class. A promising performance and generalizability across multiple datasets makes the proposed local-global context aware encoding based viral-host PPI predictor LGCA-VHPPI an appropriate candidate for other pathogens-human PPI prediction. Like viral-host PPI prediction task, a robust computational approach to accurately determine the interaction of other pathogens such as protist or bacteria (e.g Haemophilus influenza) with human protein is mainly comprised of two different modules 1) Effective statistical representations Generation 2) Interaction Prediction. This paper provides a unique way to generate an effective statistical representations of viral-host protein sequences by modeling the local and global context of residues, using which even a simple deep forest classifier manages to extract most discriminative features which help the classifier to outperform state-of-the-art viral-host PPI prediction approaches. Hence, we consider this unique way can be used to generate an effective statistical representations of bacteria-human or prostist-human proteins sequences which can be passed to any machine or deep learning classifier for accurate pathogen-human PPI prediction.

### 4.3 Performance comparison of proposed LGCA-VHPPI with existing viral-host PPI predictors over Barman dataset

Table 3 performs a fair performance comparison of proposed local-global residue context aware sequence encoding and deep forest based methodology LGCA-VHPPI with existing viral-host PPI predictors including Barman et al. [28] approach, Alguwzizani et al. [13] approach, Fatma et al. [12] approach, and Yang et al. [14] approach using benchmark Barman dataset [28] in terms of 7 distinct evaluation measures. Performance analysis of a variety of sequence encoding schemes and machine learning classifiers based viral-host PPI prediction approaches reveals that among all existing approaches, Yang et al. [14] unsupervised sequence embedding learning scheme doc2vec and Random Forest (RF) based predictor marks better performance than amino acid composition and support vector machine (SVM) based predictor as well as physicochemical properties and RF based predictor. Alguwzizani et al. [13] repeat patterns, amino acid composition based sequence encoding and SVM classifier based predictive approach marks better performance than Barman et al. [28] binary encoding and Random Forest (RF) based approach. In existing viral-host PPI predictors, primary reason of doc2vec performing better than repeat patterns and amino acid composition based sequence encoding, residue frequency based encoding, and physico-chemical properties based encoding is its aptitude to capture precise semantic relatedness of residues. Whereas physico-chemical properties and residue frequency based sequence encoding schemes mark lowest predictive performance as they neglect the relationships among various amino acid segments as a function of context of entire viral-host protein sequences.

**Table 3:** Performance comparison of proposed LGCA-VHPPI with existing viral-host PPI predictors over a benchmark Barman dataset in terms of 7 different evaluation measures. Performance figures of Barman et al. SVM [28], Barman et al. RF [28], Alguwzizani et al SVM. [13], and Yang et al. RF [14] are taken from Yang et al. [14] work.

Approach	ACC	SN	SP	PR	F1	MCC	AU-ROC
Yang et al. RF [14]	79.17	81.85	76.45	77.83	79.79	58.40	87.1
Alguwzizani et al. SVM [13]	78.6	73.72	83.48	81.69	77.50	57.50	84.70
Barman et al.’s SVM [28]	71.00	67.00	74.00	72.00	69.41	44.0	73.00
Barman et al.’s RF [28]	72.41	89.08	55.66	82.26	66.39	48.00	76.00
**Proposed LGCA-VHPPI**	**82.00**	**82.00**	**89.37**	**82.40**	**81.47**	**63.99**	**88.00**

It is evident from the Table 3 that proposed Local-Global residue context aware viral-host protein sequence encoding and deep forest model significantly outperforms a variety of viral-host PPI predictors over Barman dataset by addressing the discrepancies present in existing predictive approaches at the level of feature extraction and interaction prediction. Instead of relying solely on precise local contextual information of residues to discriminate interactive viral-host protein sequences from non-interactive ones as done by Yang et al. [14], it goes many steps further by capturing comprehensive semantic relatedness of residues, short range residue dependencies, unique residue combination distribution and complementing it with long range and positional invariance information of residues important for interaction prediction. For Barman dataset [28], LGCA-VHPPI outperforms state-of-the-art performance by the figure of 14%, 6%, 5%, 3%, and 2% with respect to specificity, MCC, precision, accuracy, and F1-score.

### 4.4 Performance comparison of proposed LGCA-VHPPI with existing viral-host PPI predictors over DeNovo dataset

Table 4 performs a detailed performance comparison of proposed local-global residue context aware sequence encoding and deep forest based methodology LGCA-VHPPI with existing viral-host PPI predictors including Alguwzizani et al. [13] approach, Fatma et al. [12] approach, and Yang et al. [14] approach using benchmark DeNovo dataset [12] in terms of 7 distinct evaluation measures. Among existing viral-host PPI predictors, on DeNovo datasets, just like Barman dataset [28], once again doc2vec and RF based viral-host predictor achieves higher performance figures followed by repeat patterns, amino acid composition based sequence encoding and SVM based predictor, and physicochemical properties and RF based predictor. However, existing viral-host PPI predictors lack of aptitude to continuously model short as well as long range dependencies of residues [14] marks room for improvement. Proposed Local-Global residue context aware viral-host protein sequence encoding captures heterogeneous residue ordinal and contextual information which eventually helps the deep forest model to identify the most crucial residue distributions for both interactive and non-interactive viral-host protein sequences. For benchmark DeNovo dataset [12], LGCA-VHPPI surpasses the previous best performance [14] by 4%, 1%, 2%, 1% in terms of sensitivity, accuracy, MCC, and F1-score.

**Table 4:** Performance comparison of proposed LGCA-VHPPI with existing viral-host PPI predictors over benchmark DeNovo dataset [12] in terms of 7 different evaluation measures. Performance figures of DeNovo SVM [12], Alguwzizani et al. SVM [13], and Yang et al. RF on DeNovo Dataset [12] are taken from Yang et al. work [14].

Approach	ACC	SN	SP	PR	F1	MCC	AU-ROC
Yang et al. RF [14]	93.23	90.33	96.17	95.99	93.07	86.60	98.10
Alguwzizani et al. SVM [13]	86.47	86.35	86.59	86.56	86.46	72.90	92.60
Fatma et al. SVM [12]	81.90	80.71	83.06	–	–	–	–
**Proposed LGCA-VHPPI**	**94.24**	**94.24**	**96.47**	**94.32**	**94.23**	**88.56**	**98.49**

### 4.5 Performance comparison of proposed LGCA-VHPPI with existing viral-host PPI predictors over Yang dataset

Table 5 performs a fair performance comparison of proposed local-global residue context aware sequence encoding and deep forest based methodology LGCA-VHPPI with Barman et al. SVM [28], Fatma et al. [12] SVM approach, and state-of-the-art Yang et al. [14] approach using benchmark Yang et al. dataset [14] in terms of 7 distinct evaluation measures. It is evident from the Table 5 that proposed LGCA-VHPPI approach significantly outperforms both Barman et al. SVM [28] and Fatma et al. SVM [12] approaches across all seven evaluation metrics such as accuracy by 4%, sensitivity by 44%, specificity by 50%, precision and F1-score by 6%, MCC by 17%, and and AU-ROC by 12%. Analysis of performance figures produced by state-of-the-art predictor [14] over Yang dataset [14] indicates that state-of-the-art predictor [14] is biased towards type II error. In contrast, the proposed LGCA-VHPPI is neither biased toward type I error nor type II error as it achieves quite consistent performance across different evaluation metrics. LGCA-VHPPI outperforms state-of-the-art predictor [14] sensitivity figure by a large value of 58%, F1-score by the value of 40%, and replicate top performance in rest of the evaluation metrics over Yang dataset [14].

**Table 5:** Performance comparison of proposed LGCA-VHPPI with existing viral-host PPI predictors over Yang dataset [14] in terms of 7 different evaluation measures. Performance Figures of Barman et al. SVM [28] and Fatma et al. SVM [12] on Yang et al. dataset are computed using their proposed methodologies. Performance figures of Yang et al. RF [14] are taken from Yang et al. work [14].

Approach	ACC	SN	SP	PR	F1	MCC	AU-ROC
Barman et al. SVM [28]	90.91	50.0	50.0	82.64	86.58	42.2	82.7
Fatma et al. SVM [12]	90.97	50.68	50.68	88.96	86.86	9.0	78.2
Yang et al. RF [14]	94.05	36.92	99.76	94.04	53.03	56.86	95.55
**Proposed LGCA-VHPPI**	**94.22**	**94.22**	**1.0**	**94.84**	**93.34**	**58.71**	**94.00**

## 5 Viral host protein protein interaction pathway analysis

Viral-host PPI prediction only facilitates whether particular viral-host protein pairs are interactive or non-interactive. The main goal of viral-host PPI pathway analysis is to determine which viral proteins interact with which host proteins. Beside providing a robust viral-host PPI predictor, we also facilitate viral-host PPI pathway analysis using proposed predictor which utilizes the interaction information of viral and host proteins. To accomplish viral-host PPI pathway analysis, we take the model trained on Denovo training set and assess the interaction pathway analysis power of trained model on a randomly chosen virus protein and human protein from test set of Denovo dataset.

**Fig 9:**
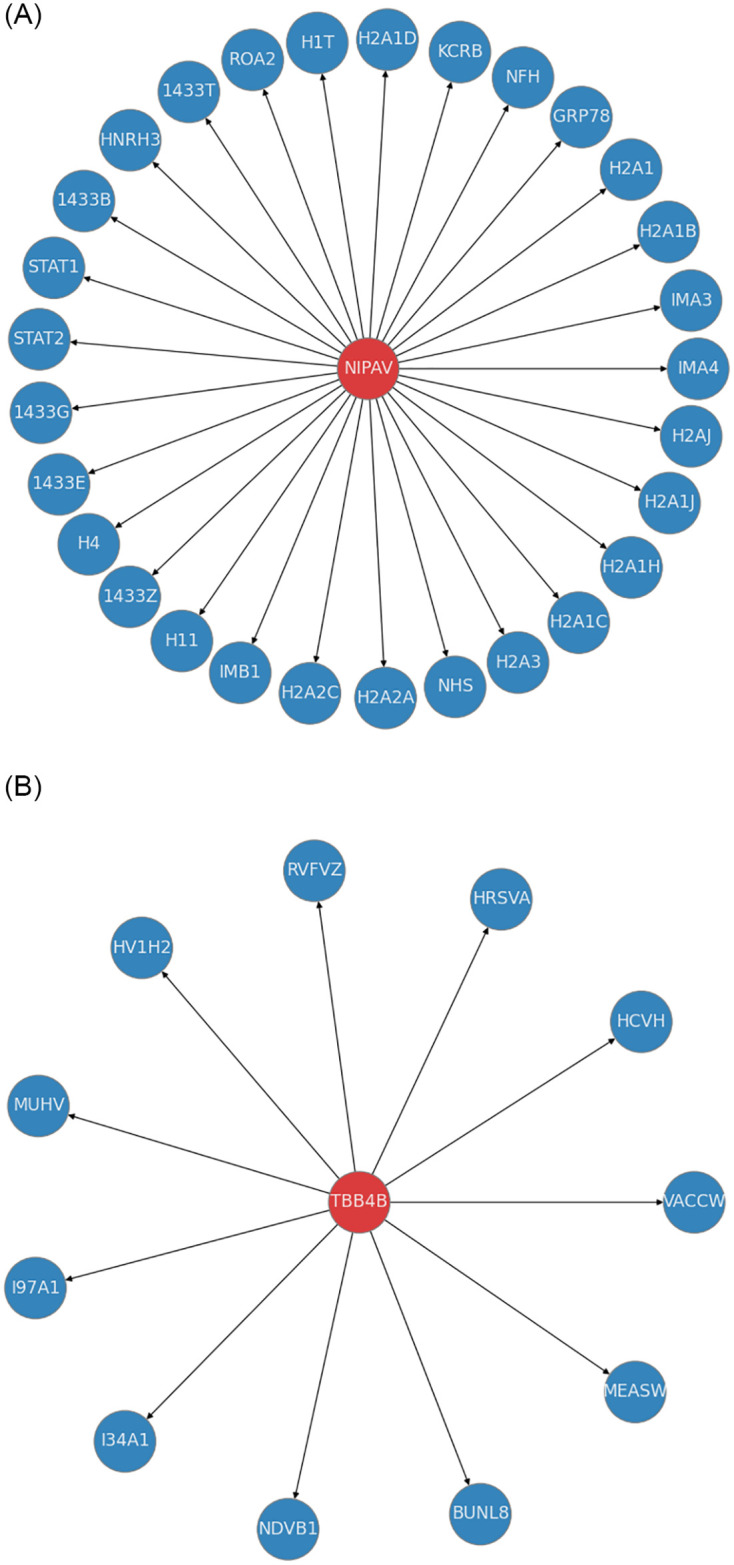
Nipah virus (Acronym: Nipav, identifier: P0C1C7) interactions with multiple human proteins and human protein Tubulin beta-4B (Acronym: TBB4B identifier: P68371) interactions with multiple virus proteins. Here only Short Names of Viruses and Human Proteins are Mentioned. Complete Details of Short names, Scientific Names, and PMIDs of Multiple Human and Virus Proteins are provided in S1 Data. (a) Virus P0C1C7 interactions with multiple human proteins. (b) Human Protein P68371 interactions with multiple viruses.

To illustrate better, interaction pathways of randomly selected viral protein called Nipah virus (Acronym: Nipav, identifier: P0C1C7) and arbitrarily selected human protein called Tubulin beta-4B (Acronym: TBB4B identifier: P68371) are shown in Fig 9(a) and 9(b) respectively. In the Fig 9(a), central node indicates the Nipah virus and surrounding associated nodes are 29 human proteins (e.g Importin subunit alpha-4 OS Histone H1t OS) which are found as interacting partners of Nipah virus in the test set. Likewise, Fig 9(b) central node represents Tubulin beta-4B human protein and surrounding associated 11 nodes (e.g Non-structural protein 1 OS = Influenza A virus, V protein OS = Measles virus, Genome polyprotein OS = Hepatitis C virus) are its interactive viral proteins partners found in the test set. The complete list of virus or human proteins used in both illustrations, together with their short names, scientific names, and PMIDs is provided in S1 Data to facilitate the readers.

Proposed model accurately predicts 29 positive interactions of Nipah virus and 11 positive interactions of Tubulin beta-4B human proteins. A similar performance trend is shown by proposed model for various other viruses and human proteins which is why it manages to achieve promising performance in terms of different evaluation metrics as compared to state-of-the-art viral-host PPI predictors. This also reveals the supreme potential of proposed model to perform comprehensive interaction pathway analysis of multiple viral species with respect to human proteins and vice versa.

## 6 An interactive and user-friendly LGCA-VHPPI web server

To facilitate Genomics and Proteomics researchers and practitioners, we have developed an interactive and user-friendly LGCA-VHPPI web server available at https://sds_genetic_analysis.opendfki.de/HVI/. This web server can be used to determine interactions among viral and host proteins merely using raw sequences related to diverse viral species. It can also be used to complement experimental approaches because it can validate experimentally detected viral-host PPIs. Unlike existing Genomics and Proteomics sequence analysis web servers, it can be used to train and optimize machine learning model from scratch on account of proteins belonging to new viral species and perform prediction on novel sequences belonging to existing or new virus species.

## 7 Conclusion

A comprehensive performance analysis of proposed viral-host PPI predictor leads to conclude that generating statistical representations of viral-host protein sequences by fusing comprehensive local and global residue contextual information proves really effective to largely raise the generalizeability of deep forest classifier across a variety of species. While local residue context aware encoder assigns higher weights to those residues which share similar context while generating statistical representations of viral-host protein sequences, global residue context aware encoder generates statistical representations of viral-host protein sequences by modelling long range dependencies and positional invariance of residues. Using optimized sequence representation based on the integration of local and global context aware encoders, proposed viral-host PPI predictor(LGCA-VHPPI) manages to extract crucial residue correlations and hidden patterns important for accurate interaction prediction. A compelling future line of current work would be to investigate the performance impact of diverse neural strategies (e.g attention) at the level of sequence encoding and interaction prediction.

## Supporting information

S1 Data (XLSX)

S2 Data (BST)
